# 

**Published:** 1892-10-01

**Authors:** 


### CONTENTS.

| | PAGE |
|---|---|
| **The Public School-Boy's Body** | 1 |
| **Passing Topics.**—A Wise Resolve | 2 |
| **Diet in Disease.** By Mrs. Ernest Hart. I.—Food and Food Values | 3 |
| **The Attitude of Great Writers Towards Disease.** XII.—Dumas on the Art of the Poisoner | 5 |
| **Medical History from the Earliest Times.** By E. T. Withington, M.A., M.B., Oxon. XXV.—Nestorian Medical Schools | 6 |
| **Annotations.**—Shall Doctors take the Lead?—Singing-Masters' Science—The Doctor, the Workshop, and the State | 7 |
| **Notes and News** | 8 |
| **The Hospital Clinic—** | |
| St. Thomas's Hospital.—Treatment of Fracture of the Patella | 9 |
| Royal Infirmary, Edinburgh.—The Treatment of the Dropsy of Bright's Disease | 11 |
| **Editor's Letter Box.**—The Miraculous Cures of Lourdes | 12 |
| **The Institutional Workshop—** | |
| Hospital Administration. III.—Special Hospitals—Children | 13 |
| Practical Departments. III.—The Laundry | 14 |
| The Cure of Poverty.—An Account of the North Kensington Friendly Workers Among the Poor. By Mrs. C. Furley Smith | 15 |
| **Scraps and Gleanings** | 16 |

### EXTRA SUPPLEMENT.—THE NURSING MIRROR.

| | |
|---|---|
| **En Passant.**—"Nursing Notes" and its Cholera Number—Derry City and County Infirmary—Dundee Royal Infirmary—Quality in Quarantine—Cigarettes and Cylinders—Macclesfield Cottage Hospital and District Nursing—On Active Service—Bazaars and Benevolence—English Invalids at San Remo—Littleborough District Nursing Association—Clifton, Bristol. Nurses' Co-operation | i |
| **Lectures for Asylum Attendants.** By William Harding, M.B. III.—Cleanliness | ii |
| **Examination Questions—Wants and Workers** | ii |
| **Neat Beds—For Reading to the Sick.**—The Nettle | iii |
| **How to Become a Nurse and How to Succeed.** By Honnor Morten | iv |
| **Hints for Nurses—Presentation** | iv |
| **Everybody's Opinion**—Oddfellows and District Nursing—Nurses' Co-operation at Clifton—Trained Nurses' Club—Food and Wages | v |
| **Notes and Queries—** | v |
| **Appointments** | v |
| **An Interesting Presentation** | v |
| **Where to Go** | v |
| **Off Duty.**—The Result of a Chill | vi |

